# 肠道菌群基于脑-肠轴影响肺癌脑转移的作用机制研究进展

**DOI:** 10.3779/j.issn.1009-3419.2025.101.20

**Published:** 2025-11-20

**Authors:** LI Dan, CHEN Liang, LV Pengqiang, YANG Quanshi, YANG Jianxin, AN Deming, YU Yu, GAO Guoming

**Affiliations:** ^1^730000 兰州，甘肃中医药大学（李丹，余雨）; ^1^Gansu University of Chinese Medicine, Lanzhou 730000, China; ^2^741020 天水，甘肃中医药大学附属天水中西医结合医院（陈亮，吕鹏强，杨全石）; ^2^Tianshui Hospital of Integrated Traditional Chinese and Western Medicine Affiliated to Gansu University of Chinese Medicine, Tianshui 741020, China; ^3^741000 天水，天水市中医医院（杨建新，安德明）; ^3^Tianshui Traditional Chinese MedicineHospital, Tianshui 741000, China; ^4^741000 天水，天水市妇幼保健院（高国明）; ^4^Tianshui Maternal and Child Health Hospital, Tianshui 741000, China

**Keywords:** 肺肿瘤, 肠道菌群, 脑转移, 脑-肠轴, 作用机制, Lung neoplasms, Gut microbiota, Brain metastasis, Brain-gut axis, Mechanism of action

## Abstract

肺癌作为全球癌症死亡的主要原因之一，其并发症脑转移（以脑实质为主）严重影响患者的生活质量及预后。传统治疗手段，如手术、放疗及化疗，虽在临床实践中取得了一定疗效，却因毒性和患者耐受性问题而应用受限。新兴的靶向治疗与免疫治疗凭借较少的副作用展现出显著疗效，但仍然面临部分耐药及基因突变等挑战。近年来，肠道菌群与肺癌脑转移之间的关联性研究成为新热点，其作用机制可能通过脑-肠轴介导的神经内分泌-免疫多维度交互途径对肺癌脑转移进程产生影响。本文综述了肠道菌群基于脑-肠轴调控肺癌脑转移的免疫调节、内分泌代谢调节、神经递质调控等具体作用机制，及调节肠道菌群在肺癌脑转移中的治疗和传统医学研究，旨在为肺癌脑转移的防治策略提供理论支撑和临床潜在治疗靶点。

## Abstract (en)

Lung cancer, one of the leading causes of cancer-related deaths worldwide, severely influences patients' quality of life and prognosis due to its complication of brain metastasis (primarily involving the brain parenchyma). While traditional treatments such as surgery, radiotherapy, and chemotherapy have demonstrated efficacy in clinical practice, their application remains limited due to toxicity and patient tolerance issues. Emerging targeted therapies and immunotherapies, which exhibit significant efficacy with fewer side effects, still face challenges including partial drug resistance and genetic mutations. In recent years, research on the association between gut microbiota and lung cancer brain metastasis has gained prominence. Its mechanism of action may influence the progression of brain metastasis through multidimensional interactions via the brain-gut axis, involving neuroendocrine and immune pathways. This review examines specific mechanisms by which gut microbiota modulate lung cancer brain metastasis through immune regulation, endocrine-metabolic regulation, and neurotransmitter control via the brain-gut axis, as well as therapeutic and traditional medicine research on regulating gut microbiota in lung cancer brain metastasis. It aims to provide theoretical support and identify potential clinical therapeutic targets for the prevention and treatment of lung cancer brain metastasis.

肺癌已成为全世界各国发病率和死亡率最高的恶性肿瘤之一，而中国的发病率和死亡率均高于美国和其他国家^[1]^。据国家癌症中心数据^[2]^统计，2022年中国肺癌新发病例高达106.06万，占全部恶性肿瘤的22.0%，死亡人数73.33万，占全部恶性肿瘤死亡的28.5%。肺癌患者整体预后较差，5年生存率低，这可能受早期诊断条件和治疗理念的影响。脑转移是肺癌严重的并发症，脑实质作为脑转移的最主要亚型，好发于大脑半球，其次为小脑和脑干，主要表现为头痛、呕吐和视神经乳头水肿，而脑膜转移发生率虽低，但预后更差，且脑脊液鉴别存在一定难度，因此脑实质转移仍是当前肺癌脑转移研究的重点方向^[3]^。流行病学研究^[4]^显示，约50%的非小细胞肺癌（non-small cell lung cancer, NSCLC）患者在病程中可能发生脑转移，其不仅是NSCLC患者复发和死亡的主要原因之一，还使得肺癌脑转移的治疗和管理难度明显增大。既往对肺癌脑转移的研究都聚焦在基因突变、上皮间质转化（epithelial-mesenchymal transition, EMT）等肿瘤内在特性方面，而近期的研究^[5]^发现，肠道菌群可通过脑-肠轴介导的神经内分泌-免疫系统等多途径调控肺癌脑转移进程，其代谢产物、菌体成分及信号分子可能通过影响远端器官功能，进而参与脑转移调控。

## 1 肠道菌群概述

### 1.1 肠道菌群在疾病中的演变

肠道菌群研究已从早期微生物形态观察、单一疗效探索，逐步发展为整合多组学技术的生物学前沿^[6]^。脑-肠轴理论揭示微生物代谢物通过神经内分泌途径调控大脑功能的机制^[7]^。阿尔茨海默病模型小鼠研究证实肠道菌群失调可促进脑内β淀粉样蛋白沉积^[8]^，而粪菌移植（fecal microbiota transplantation, FMT）能通过调节小胶质细胞活性逆转病理改变^[9]^，这说明肠道菌群的失调会影响疾病的发展。另有研究^[10]^发现肠道菌群代谢物丁酸具有抗肿瘤作用，且血清丁酸含量与NSCLC患者的抗程序性细胞死亡受体-1（programmed cell death protein-1, PD-1）疗效有关，这一发现为微生物组免疫疗法提供理论依据。综上，肠道菌群在疾病中的研究正经历从描述性观察到机制性解析，从局部影响到全身调控，都体现了其在疾病的发生发展中起着重要的作用。

### 1.2 肠道菌群的组成与功能

肠道菌群由细菌、病毒、真菌等多元微生物共同构成，其组成与功能和宿主的健康紧密关联^[11]^。细菌中厚壁菌门和拟杆菌门的比例影响短链脂肪酸（short-chain fatty acids, SCFAs）的生成^[12]^。此外，变形菌门、放线菌门等在免疫调节中发挥关键作用^[13]^。病毒以噬菌体为主，通过调控细菌群落结构间接影响宿主代谢^[14]^。真菌可参与宿主自身免疫调节，其中白色念珠菌、马拉色菌过度增殖易诱发肠道炎症^[15]^。

生理功能上，肠道菌群通过代谢膳食纤维生成SCFAs，为结肠上皮细胞供能并调节宿主脂质代谢与能量平衡^[16]^。菌群及其代谢产物经抗原呈递细胞（antigen-presenting cells, APCs）表面的Toll样受体（Toll-like receptors, TLRs）识别，同时损伤相关分子（damage-associated molecular patterns, DAMPs）亦可激活该类细胞，进而诱导调节性T细胞（regulatory T cells, Tregs）的分化。同时抑制过度炎症反应，促进抗体产生与免疫记忆形成^[17]^，还能分泌黏液层重塑酶、抗菌肽等维持肠道屏障完整性^[18]^。此外，菌群还参与维生素合成及神经递质调控，形成“微生物-肠-脑轴”等跨器官模式以维持宿主生理稳态^[19]^。

## 2 脑-肠轴概述

### 2.1 脑-肠轴核心通路

脑-肠轴通过神经内分泌-免疫系统的协同作用，实现肠道活动与大脑双向动态交互。肠道菌群代谢产物、炎症信号等可经由外周神经或内分泌通路影响大脑情绪、认知及行为，而大脑则通过自主神经系统（autonomic nervous system, ANS）和神经内分泌系统调控肠道运动、分泌及菌群平衡，形成脑-肠轴的闭环^[20]^。该轴的核心通路包括神经通路、内分泌调节通路、免疫信号通路，该轴可介导肠道微生物群对大脑等远端器官形成调控。在神经通路中，迷走神经通路作为核心通路结合肠神经系统（enteric nervous system, ENS）及ANS形成功能网络。肠道菌群的代谢产物SCFAs、神经递质前体或炎症信号，可被嗜铬细胞感知，通过迷走神经传入中枢，中枢通过ANS反向调控肠道运动、屏障功能及菌群平衡^[21,22]^。ENS作为ANS的重要组成部分，可独立控制肠道功能，包括运动活动、分泌、吸收和免疫防御，在维持肠道稳态以及与微生物群和宿主系统相互作用方面发挥着关键作用^[23]^。在内分泌通路中，以下丘脑-垂体-肾上腺（hypothalamic-pituitary-adrenal axis, HPA）轴为核心调控中枢，菌群失调可激活HPA轴，促使下丘脑室旁核分泌促肾上腺皮质激素释放激素，刺激垂体分泌促肾上腺皮质激素，进而诱导肾上腺释放糖皮质激素（glucocorticoid, GC），该激素通过血液循环作用肠道，抑制肠道屏障功能并调节菌群的平衡^[24]^。肠道内分泌细胞嗜铬细胞经肠道菌群的刺激分泌肠促胰素、5-羟色胺（5-hydroxytryptamine, 5-HT）、胆囊收缩素（cholecystokinin, CCK）等激素，这些激素经血液循环作用于中枢神经系统，从而形成双向调节^[25]^。免疫信号通路中肠道菌群通过肠道相关淋巴组织激活APCs，诱导Tregs、辅助性T细胞（T-helper cell, Th1）、细胞毒性T淋巴细胞（cytotoxic T lymphocyte, CTL）等细胞分化与活化，分泌白细胞介素-10（interleukin-10, IL-10）、转化生长因子-β（transforming growth factor-β, TGF-β）、干扰素-γ（interferon-γ, IFN-γ）等细胞因子，维持肠道屏障完整性及免疫功能^[26]^。肠道菌群失调引发局部炎症产生的炎症因子可经血液循环诱导脑部小胶质细胞活化，而大脑释放的神经肽亦可调节肠道免疫细胞功能，形成免疫通路的双向调控^[27]^。

### 2.2 脑-肠轴与肿瘤的关联

脑-肠轴作为连接中枢神经系统与肠道微生物之间的双向调节通路，近年来在肿瘤发生、发展及治疗中起着重要的作用，其失衡状态为肿瘤细胞的生长与增殖提供了关键条件，机制涉及神经内分泌调节、代谢产物调控、炎症反应等多方面^[28]^。（1）神经内分泌层面：慢性应激会激活经典的神经内分泌系统HPA轴和交感神经系统（sympathetic nervous system, SNS），HPA轴和SNS激活过程中产生的GC、儿茶酚胺等应激激素可以通过多种机制促进肿瘤发生与进展^[29]^。而肠道菌群可能有助于预防应激激素相关的肿瘤进展^[30]^。（2）免疫层面：肠道微生物群作为脑-肠轴调控机体免疫的核心枢纽，其失衡可引发免疫抑制微环境，进而促进肿瘤发展^[31]^。SCFAs等代谢产物通过促进初始T细胞向Tregs细胞的分化来减轻炎症、抑制选定的组蛋白去乙酰化酶（histone deacetylases, HDACs）发挥表观遗传作用等多种机制改变细胞基因表达，影响着包括肿瘤在内的多种疾病的发生和进展^[32,33]^。（3）代谢层面：肠道微生物群的代谢产物是脑-肠轴连接肠道与全身组织的关键“信使”，其成分改变可直接或间接调控肿瘤发生。除SCFAs的免疫调节作用外，失调菌群产生的次级胆汁酸脱氧胆酸可通过诱导肠道上皮细胞DNA损伤、激活核因子-κB（nuclear factor-κB, NF-κB）信号通路，直接促进细胞恶性转化，其与结直肠癌的发生密切相关^[34]^。（4）炎症层面：免疫系统与肠道菌群相互作用以维持肠道稳态，而菌群失调会促进慢性炎症和肿瘤的发生。肠道微生物及其有毒代谢物可能经循环系统迁移，导致宿主生理状态失衡，并通过肠-脑轴、肠-肝轴和肠-肺轴分泌各种神经活性分子，影响特定器官的炎症和肿瘤发生^[35]^。持续的炎症状态会激活丝裂原活化蛋白激酶（mitogen-activated protein kinase, MAPK）/细胞外信号调节激酶（extracellular signal-regulated kinase, ERK）、磷脂酰肌醇3-激酶（phosphatidylinositol 3-kinase, PI3K）/蛋白激酶B（protein kinase B, AKT）等信号通路，促进肿瘤细胞增殖、抑制凋亡，同时加速炎症细胞向病灶聚集，形成“炎症-肿瘤”恶性循环^[36]^。

## 3 脑-肠轴对肺癌脑转移靶向治疗及免疫治疗的影响

脑-肠轴通过肠道菌群介导的代谢调控影响肺癌脑转移的靶向治疗疗效。研究表明，肺癌脑转移高度依赖肿瘤细胞突破血脑屏障（blood-brain barrier, BBB）的能力，而靶向治疗的核心在于药物能否穿透BBB^[37]^，而调节某些肠道菌群及代谢物可加强BBB的通透性，从而提升脑转移灶的靶向治疗血药浓度^[38]^。在免疫治疗中，肠道菌群调节可作为免疫检查点抑制剂（immune checkpoint inhibitors, ICIs）的“增敏剂”调控全身抗肿瘤免疫应答。Preet等^[39]^研究发现双歧杆菌分泌的细胞外囊泡通过动力蛋白依赖性内吞作用被肺癌细胞摄取，激活TLR4/NF-κB通路上调程序性细胞死亡配体1（programmed cell death ligand 1, PD-L1）表达，同时穿透肠道屏障到达肿瘤组织，与抗PD-1疗法协同增强抗肿瘤免疫反应，增加CD8^+ ^T细胞浸润，抑制肿瘤生长。同时，脑-肠轴有助于改善脑内微环境，使其产生免疫应答以对抗肺癌的脑转移、克服其免疫耐药机制^[40]^。

## 4 肠道菌群基于脑-肠轴调控肺癌脑转移潜在核心机制

### 4.1 免疫调节机制

肺癌脑转移灶的形成依赖于脑内免疫抑制微环境的构建，而肠道菌群在此过程通过脑-肠轴远程调控^[41]^。肠道菌群通过促进免疫细胞的增殖与活化，提升外周血中免疫效应细胞向脑部实质的募集能力，加强对脑转移细胞的免疫监视^[42]^。这与肺癌脑转移患者外周血中Tregs、自然杀伤（natural killer, NK）细胞活化异常、巨噬细胞、CTL功能受损的标志性免疫指标变化一致^[43]^。而这些免疫细胞释放的细胞因子可通过血液循环为肺癌的脑转移提供定植条件。研究发现双歧杆菌的成分胞外多糖（exopolysaccharide, EPS）通过TLR2/髓样分化因子88（myeloid differentiation factor 88, MyD88）依赖的方式被树突状细胞（dendritic cell, DC）感知，诱导IL-12和肿瘤坏死因子-α（tumor necrosis factor-α, TNF-α）的产生，激活Th1细胞及CTL^[44,45]^。这提示有益菌可能通过逆转免疫抑制状态延缓肺癌脑转移。相反，菌群的失调可诱导IL-6、TNF-α等促炎因子释放使得髓源性抑制细胞（myeloid-derived suppressor cells, MDSCs）扩增及Tregs比例升高，形成免疫抑制作用^[46]^。研究^[47]^证明，肺癌脑转移A549-F3细胞通过IL-6/Janus激酶2（Janus kinase 2, JAK2）/信号转导和转录激活因子3（signal transducer and activator of transcription 3, STAT3）信号通路诱导M2型小胶质细胞，而极化的M2型小胶质细胞通过影响转移细胞的定植来促进NSCLC脑转移的发展。低浓度的SCFAs代谢产物丁酸可能通过上调长链非编码RNA H19促进M2型巨噬细胞极化与功能来促进肺癌的脑转移^[48]^。丁酸具有双重免疫调控反应，不仅调节Tregs的分化，还能通过分泌细胞因子IL-10和IL-13上调肿瘤相关巨噬细胞（tumor associated macrophages, TAMs）的特异性标志物精氨酸酶-1（arginase-1, Arg-1），从而促进M2型巨噬细胞极化。M2型的极化为抵达脑部的肺癌脑转移细胞提供一个免疫重塑的“转移前微环境”。综上，肠道菌群基于脑-肠轴调节免疫细胞活性、抑制免疫抑制环境、调节小胶质细胞向M2型极化等途径直接或者间接地影响肺癌脑转移。

### 4.2 内分泌代谢调节

肠道菌群及代谢产物构成一个复杂的“内分泌代谢器官”，其基于脑-肠轴与肺癌脑转移相联系。研究^[49]^发现生理浓度的SCFAs丁酸通过表观遗传抑制组蛋白去乙酰化，增强血管内皮细胞间紧密连接蛋白（claudin-5及occludin）的表达，从而增强BBB的完整性。去乙酰化这一过程能抑制NF-κB通路的激活，间接避免破坏紧密连接蛋白而影响BBB的稳定性^[50]^。同时，丁酸还可激活G蛋白偶联受体41/43（G protein-coupled receptor 41/43, GPR41/43），该信号通路可协同加强BBB的保护性，以降低其通透性，促进CD8^+^ T细胞向脑转移灶的浸润，抑制M2型的极化，从而发挥抗肿瘤作用^[51,52]^。这两条通路协同加强BBB的完整性，有效抑制肺癌脑转移细胞的穿透能力。与此同时，肠道菌群通过调控全身性的代谢与内分泌状态，间接影响肺癌脑转移的进程。随着肠道菌群及代谢物水平及丰度下降，其对巩固肺癌脑转移的BBB和激活肿瘤免疫的作用被抑制^[53]^。肠道菌群也通过脑-肠轴神经信号对肺癌的脑转移进行调控。丁酸通过抑制HPA轴的过度激活，促进Tregs的分化、增强抗炎因子IL-10的表达，从而减少皮质醇等激素的过度释放，以间接减少炎症和保护BBB完整性^[54]^。综上，以肠道菌群及代谢产物作为媒介，脑-肠轴通过内分泌代谢途径重塑全身状态，调节免疫细胞及保护BBB影响肺癌的脑转移。SCFAs调节机制见图1。

**图 1:**
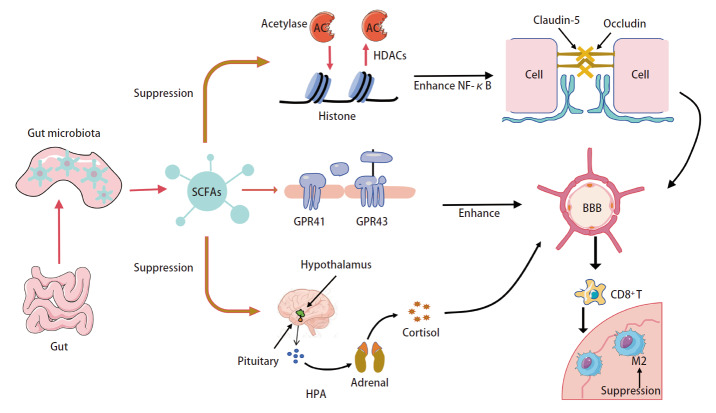
SCFAs调节机制图

### 4.3 神经递质调控

肠道菌群通过脑-肠轴调控神经递质的合成影响肺癌脑转移的微环境重塑，为脑转移提供脑内细胞定植环境。神经递质作为脑-肠轴关键效应分子，通过重塑脑内微环境调控肺癌脑转移细胞的侵袭与定植，成为连接肠道菌群与肺癌脑转移的关键通路^[55]^。γ-氨基丁酸（gamma-aminobutyric acid, GABA）是中枢神经系统主要的抑制性神经递质，其稳态失衡与神经炎症、肿瘤进展密切相关。由乳酸杆菌合成的神经递质，经血液循环跨越BBB进入中枢，可补充中枢GABA池^[56]^。Xie等^[57]^在实验研究中发现，通过下调肺癌脑转移倾向的肿瘤细胞中的叉头框蛋白A2（forkhead box A2, FOXA2）表达来抑制4-氨基丁酸转氨酶（4-aminobutyrate aminotransferase, ABAT），从而上调GABA的表达，而GABA通过激活NF-κB通路并协同星形胶质细胞为NSCLC脑转移提供免疫抑制微环境且促进肿瘤细胞定植。5-HT作为必需氨基酸色氨酸代谢而来的中枢神经递质，对癌细胞增殖、侵袭、播散和肿瘤血管生成具有潜在的刺激作用^[58]^。梭菌属的梭状芽孢杆菌通过刺激肠嗜铬细胞从而增加肠道5-HT水平^[59]^。Sun等^[60]^实验研究发现肠道菌群中的5-HT通过抑制小核糖核蛋白多肽G（small nuclear ribonucleoprotein polypeptide G, SNRPG）表达，使Wilms肿瘤1（Wilms tumor 1, WT1）积累，WT1结合细胞周期蛋白依赖性激酶14（cyclin-dependent kinase 14, CDK14）启动子激活转录，从而促进NSCLC细胞迁移、侵袭及转移。多巴胺（dopamine, DA）是调控肿瘤细胞增殖、迁移的重要神经递质，其功能紊乱与肿瘤侵袭、转移密切相关^[61]^。肠道菌群是DA合成和调节的重要参与者，其平衡状态直接影响着DA的分泌^[62]^。Wu等^[63]^通过体外实验表明，DA受体D2（DA receptor D2, DRD2）的过表达通过下调NF-κB信号通路抑制大肠杆菌脂多糖（lipopolysaccharide, LPS）诱导的NSCLC细胞系A549和SK-MES-1细胞增殖和生长。综上，肠道菌群通过调节脑-肠轴关键信号分子调控肺癌脑转移。

## 5 调节肠道菌群在肺癌脑转移中的治疗及传统医学研究

肠道菌群失衡是肺癌脑转移的关键调控因素，益生菌的干预、FMT等在临床展现出显著的潜力。Liu等^[64]^的研究通过宏基因组学和靶向代谢组学分析，发现NSCLC脑转移患者与无远处转移患者的肠道菌群组成存在显著差异。脑转移患者微生物多样性更高，且富集拟杆菌属等特定菌属。代谢组学显示，与免疫调节和血管通透性相关的SCFAs及血管紧张素（angiotensin, Ang）等代谢物水平改变，可能影响脑转移发展。机器学习模型识别出杆菌、N-乙酰-L-谷氨酸（N-acetyl-L-glutamate, NAG）等生物标志物，可用于脑转移早期检测，这说明肠道菌群失调及其代谢产物可能促进肺癌脑转移。NSCLC患者肠道微生物多样性降低，群落结构改变。脑转移阶段，梭杆菌门和变形菌门病原体增加，厚壁菌门和放线菌门的SCFAs产生菌减少。由此可见，益生菌制剂靶向补充有益菌群已成为肺癌脑转移辅助治疗的关键。而此研究^[65]^进一步证实了NSCLC患者粪便中SCFAs总浓度显著降低，尤其脑转移患者低于早期患者和健康对照组，并且乙酸和丁酸水平也呈类似趋势。此外，粪杆菌属、双歧杆菌属等特定肠道菌群组合可区分早期NSCLC患者与健康对照，以此作为诊断标志物的潜力。由此说明，通过补充有益益生菌、FMT转移等可重建患者肠道微生态平衡，为肺癌脑转移提供新型治疗策略。

中医药以“肺与大肠相表里”理论为核心，为肺癌从肠论治提供了重要的理论支撑^[66]^。肠道菌群与肺癌转移之间的关联，可借助“肺肠同治”这一中医理论建立联系。Li等^[67]^通过建立小鼠Lewis肺癌模型研究，结果表明中药复方双参颗粒可通过富集菌群中的阿克曼菌、YL32型乳酸梭菌、罗伊氏乳杆菌及其代谢产物，调控TAMs极化并抑制NF-κB通路表达来抑制肺癌脑转移。蒋等^[68]^通过实验研究发现，中药健脾固肠方与化疗联合使用通过丰富肺癌小鼠肠道菌群多样性、调节肠道Th17/Tregs细胞平衡以改善机体免疫，达到抑制肺癌小鼠肺癌细胞侵袭及转移的疗效。

## 6 小结与展望

肠道菌群通过脑-肠轴影响肺癌脑转移的作用机制已经有了初步研究，主要涉及免疫调节、内分泌代谢调节、神经递质等。虽然目前该理论为肺癌脑转移治疗提供临床指导并显现初步的支持，但仍然存在诸多不足：目前研究多聚焦于以脑实质为主的脑转移，多数的研究仍集中在肠道菌群与肿瘤的关联性分析、对具体分子机制的研究不够深入，且基于脑-肠轴的临床研究较少、多数为体外细胞实验、动物实验及临床前研究，缺乏大规模临床试验验证肠道菌群基于脑-肠轴干预肺癌脑转移的疗效与安全。且目前针对脑膜转移的机制与临床研究匮乏、无法解释其与脑实质转移的特异性。此外，肠道菌群生物利用度较低、组成存在个体差异，精准识别与肺癌脑转移相关的敏感菌群特征仍是个性化治疗的难题。综上，未来应该深化研究具体分子机制与肠道菌群相关肿瘤细胞的直接作用靶点，解析脑-肠轴信号通路的动态变化，提升肠道菌群生物利用度，筛选精准作用靶点与菌群对接，减少个体化差异。开展基于脑-肠轴的肺癌脑膜转移机制研究，明确其与脑实质转移的异质性。开展多中心FMT临床试验、结合单细胞测序与空间转录组等研究，进一步评估益生菌、FMT等干预措施对肺癌脑转移患者的疗效和安全性，最终制定基于菌群特征的精准治疗方案，以实现肺癌脑转移个体化精准治疗。
